# The Effects of Drought and Shade on the Performance, Morphology and Physiology of Ghanaian Tree Species

**DOI:** 10.1371/journal.pone.0121004

**Published:** 2015-04-02

**Authors:** Lucy Amissah, Godefridus M. J. Mohren, Boateng Kyereh, Lourens Poorter

**Affiliations:** 1 Council for Scientific and Industrial Research—Forestry Research Institute of Ghana, KNUST, Kumasi, Ghana; 2 Forest Ecology and Forest Management Group, Wageningen University, Wageningen, The Netherlands; 3 College of Agriculture and Natural Resources, Kwame Nkrumah University of Science and Technology, Kumasi, Ghana; INRA—University of Bordeaux, FRANCE

## Abstract

In tropical forests light and water availability are the most important factors for seedling growth and survival but an increasing frequency of drought may affect tree regeneration. One central question is whether drought and shade have interactive effects on seedling growth and survival. Here, we present results of a greenhouse experiment, in which seedlings of 10 Ghanaian tree species were exposed to combinations of strong seasonal drought (continuous watering versus withholding water for nine weeks) and shade (5% irradiance versus 20% irradiance). We evaluated the effects of drought and shade on seedling survival and growth and plasticity of 11 underlying traits related to biomass allocation, morphology and physiology. Seedling survival under dry conditions was higher in shade than in high light, thus providing support for the “*facilitation hypothesis*” that shade enhances plant performance through improved microclimatic conditions, and rejecting the *trade-off hypothesis* that drought should have stronger impact in shade because of reduced root investment. Shaded plants had low biomass fraction in roots, in line with the *trade-off hypothesis*, but they compensated for this with a higher specific root length (i.e., root length per unit root mass), resulting in a similar root length per plant mass and, hence, similar water uptake capacity as high-light plants. The majority (60%) of traits studied responded independently to drought and shade, indicating that within species shade- and drought tolerances are not in trade-off, but largely uncoupled. When individual species responses were analysed, then for most of the traits only one to three species showed significant interactive effects between drought and shade. The uncoupled response of most species to drought and shade should provide ample opportunity for niche differentiation and species coexistence under a range of water and light conditions. Overall our greenhouse results suggest that, in the absence of root competition shaded tropical forest tree seedlings may be able to survive prolonged drought.

## Introduction

Seedling establishment, growth and survival play an important role in forest dynamics, as they determine the future composition of the forest canopy [1]. Seedling growth and survival are influenced by resource availability (i.e. light, water and nutrients) and disturbance (fire, logging, herbivores and pathogens) [2, 3]. In tropical forests light [4] and water availability [5, 6] are spatially and temporally heterogeneous and this heterogeneity provides a productivity gradient [7]. At the upper end of the productivity gradient light is the most limiting resource, whereas at the lower end of the productivity gradient water is the most limiting resource [7]. For instance, in moist forests due to high moisture availability there is high productivity which leads to the formation of dense vegetation and results in a reduction in the amount of light that reaches the forest understorey. On the contrary, in drier forests where water is the most limiting resource there is low productivity which leads to the formation of a less dense forest canopy with more light reaching the forest understorey.

Seedling tolerance to limiting resources determines in part the composition and distribution of species. Over the past decades tropical forests have experienced a reduction in annual rainfall and in some areas, such as the Amazon forests, severe droughts have occurred [8, 9]. Climate change scenarios predict that the intensity and frequency of drought will even increase further in the near future [10–13] and drought will be the main aspect of global change which will determine the future of moist tropical forests [5, 14]. Understanding species response to drought is therefore critical in forecasting possible impacts of climate change on tree regeneration in the forest and species distribution. In lowland tropical forest, species drought performance, tolerance and the length of dry season have been found to be important determinants of species distribution [15–18]. The drought performance of a seedling may be influenced by the location (understory or gaps) where the seedling grows in the forest (i.e., its shade tolerance) [19]. The interaction between drought and shade may play an important role in how species establish, grow and survive, but information on the combined effects of drought and shade in the tropics is surprisingly scarce (but see [19–21]).

Three contrasting views have been proposed on whether there is a trade-off between shade tolerance and drought tolerance. The *trade-off hypothesis* predicts a trade-off between shade and drought tolerance, because shaded plants invest in leaves to enhance light capture, but this comes at the expense of allocation to water acquiring roots, thus making shaded plants more sensitive to drought [22]. According to the *facilitation hypothesis* drought has a weaker impact on plants in shade, because of lower air temperatures and vapour pressure deficits in shaded microsites [23, 24]. Alternatively shade and drought tolerance are uncoupled and vary independently (the *uncoupled hypothesis*) across forest and shrub species [7, 24–27]. All plants require light, water and nutrients for their survival, growth and reproduction [28]. Consequently reduced availability in any one of these resources may lead to reduced survival and growth (*resource limitation hypothesis*). Plants maximise their surface area to capture the most limiting resource (*Brouwer’s hypothesis*) [29]. Differential capacity of plants species to maximise resource capture will be of significant ecological advantage in the face of global climate change.

Functional traits may confer tolerance to shade and drought in several distinct ways [30]. There are three main mechanisms for dealing with drought stress: (1) drought avoidance (2) drought delay and (3) physiological drought tolerance [27, 31–33]. Traits that are associated with drought avoidance include leaf shedding in deciduous species to reduce water loss. Evergreen species may also delay drought by increased below ground biomass allocation, construction of fine roots, and deep roots to enhance water access and uptake [27, 32]. Evergreen plants could also produce thick leaves and reduce stomatal conductance to minimise transpirational water loss [34]. Physiological drought tolerance is defined by a plant’s ability to continue to function physiologically at low water availability (tolerance to low tissue water status). At low water potentials, drought tolerant species reduce the risk of xylem cavitation (through having dense stem with narrow vessels and pit pores), which allows them to maintain gas exchange and hydraulic conductivity [27, 31]. Additionally, species that efficiently close their stomata can tolerate dry conditions. In some coniferous species high levels of the hormone abscisic acid influences the efficient closure of the stomata during water stress [35].

Shade-grown plants typically invest in high above ground biomass (leaves and stem) and also make thin leaves to optimise light capture and utilisation [36–38]. At the whole-plant level shade-tolerant species also display their leaves efficiently to enhance light capture in a low light environment [36]. Plants grown in high light allocate relatively less to leaves and allocate more to roots to capture water and nutrients to sustain the high transpiration rates and growth rates [39, 40]. These intraspecific responses of plants to shade are also mostly found in inter specific responses to shade among species. In spite of distinct strategies under shade and drought it is possible to have traits that confer both drought and shade tolerance in plants within a conservation resource strategy [41, 42]. In such a situation shade tolerant plants will have low allocation to leaves, high allocation to stem and relatively high allocation to below ground tissues.

Phenotypic plasticity is the capacity of a genotype to alter its phenotype under changing environmental conditions [43]. Such plasticity may be of paramount importance for species to adjust to temporal and spatial variation in resource availability, but few studies have demonstrated to what extent plasticity is really associated with tolerance to shade and drought. In a study [41] with eight Mediterranean woody tree species, it was found that phenotypic plasticity was negatively related to shade tolerance (quantified as survival in deep shade), and not related to drought tolerance (quantified as survival under drought). In another study of 16 tropical rain forest shrub species in three light environments, it was found that species, which specialise for gaps had higher plasticity than understory species [44]. In gaps there is a predictable decrease in irradiance over time when the gap vegetation regrows and plants respond plastically to these changes [44]. Plasticity may be either adaptive or non-adaptive. If plasticity is adaptive, then a high plasticity would lead to higher survival under stressful conditions [45].

In this study we evaluated in a controlled cross-factorial experiment the effects of light and drought on the performance, morphology and physiology of 10 Ghanaian tree species. We addressed three research questions: (1) what are the effects of drought and shade on seedling survival and growth? (2) What are the effects of drought and shade on underlying seedling traits, such as biomass allocation, morphology and physiology? (3) How does morphological plasticity relate to survival under stressful conditions and growth under optimal conditions? We hypothesised that (1a) with decreased resource availability (light, water) there will be decreased survival and growth (the *resource limitation hypothesis*,[46]), (1b) shade will result in increased drought survival compared to high light (the *facilitation hypothesis*, [24]), (2) plants will invest in the organ that captures the most limiting resource (*Brouwer’s hypothesis*) [29], (3) higher plasticity in plant functional traits should lead to high survival under stressful conditions [45] and faster growth under optimal condition should lead to higher plasticity because plants have more carbon at their disposal to make plastic adjustment [43,44].

## Materials and Methods

### Species and study site

This study is part of a larger experiment in which we evaluated drought survival of 24 tropical tree species, and related it to their functional traits. For the present study we focused on a subset of ten tropical forest species for which we were able to evaluate their acclimatisation responses to shade and drought. The 10 species come from 8 families with different distributions (moist, wet and dry forests) in Ghana’s forests (Table 1). The species had different light requirements for regeneration: one pioneer, two shade tolerant and seven non-pioneer light demanders [47]. The species were selected because of their importance for timber trade in Ghana, their use as medicinal plants, and because of their different rainfall distributions. Seeds of the species were collected from two forest types: moist forest (Bobiri Forest Reserve), which lies between latitudes 6° 39’ and 6° 44’N and longitudes 1° 15’ and 1° 23’W and dry forest (Afram Headwaters Forest Reserve), which lies between latitudes 6° 45 'N and 7° 25' N and longitudes 1° 32' W and 1° 48' W. Permission for seed collection was granted by the Forestry Research Institute of Ghana. The responses of the selected species to drought and shade were evaluated in 4 neutral shade greenhouses located at the Forestry Research Institute of Ghana (FORIG- latitude 06° 41’N and longitude 01° 28’W). The size of each greenhouse was 5.60 m x 4.96 m wide and 3 m high and inter-greenhouse distance was 4 m. A distance of 4 m was chosen so that neighbouring greenhouses would not shade each other. The green houses were aligned north-south to ensure that they all received the same amount of light at any point in time. Each greenhouse was covered with a plastic shelter, to avoid the entry of rain in the greenhouse.

**Table 1:** List of ten tree species, their family, natural distribution and light requirements for regeneration (species guild).

Species	Family	Forest type	Species guild	Averageseed dry mass
*Entandrophragma angolense* (Welw). DC	Meliaceae	Moist forest	NPLD	0.4
*Turraeanthus africanus* (Welw. ex C.DC.) Peller	Meliaceae	Moist forest	Shade tolerant	1.0
*Piptadeniastrum africanum* (Hook.f.) Brenan	Mimosaceae	Moist forest	NPLD	0.18
*Ceiba pentandra* (Linn.) Gaertn.	Bombaceae	Dry forest	Pioneer	0.05
*Albizia zygia* (DC.) J.F. Macbr.	Mimosaceae	Dry forest	NPLD	0.06
*Pericopsis elata* (Harms) Van Meeuwen	Papilionaceae	Dry forest	NPLD	0.19
*Sterculia rhinopetala* K. Schum	Sterculiaceae	Dry Forest	NPLD	0.40
*Aningeria robusta* (*Pouteria aningeri*) *(A*. *Chev*.*)Aubrev*. *and Pellegr*.	Sapotaceae	Dry Forest	NPLD	0.75
*Antiaris toxicaria* Leschenault	Moraceae	Ubiquitous	NPLD	1.67
*Strombosia pustulata* J. Leonard	Olacaceae	Ubiquitous	Shade tolerant	1.25

NPLD = non-pioneer light demander. Species guild and preference for a different forest type is based on [49–51]. Average dry seed mass is based on [52–58].

Irradiance levels of 5% (2 greenhouses) and 20% (2 greenhouses) of full sunlight were created by using bamboo slats, mosquito netting and raffia mats. The irradiance levels were determined through daily measurements with a light meter (Fisher Scientific Traceable Dual Display light meter, Fisher Scientific, Pittsburgh, USA) for a month. Concurrent measurements of irradiance levels inside and outside of the greenhouses were made in the morning (8:00–9:00am), around noon (12:00–1:00pm) and in the afternoon (4:00–5:00pm). Irradiance level in each greenhouse was calculated as a percentage of irradiance outside the greenhouse. Daily average irradiance levels were calculated for each greenhouse. A monthly average was also calculated and was used as the irradiance level in each greenhouse. Twenty percent irradiance (20%) was used as this is typical for large forest gaps. Additionally, it has been found that seedlings of Ghanaian tree species are able to achieve maximum growth at irradiances of between 10% and 44% [48]. Hence, 20% irradiance allows the comparison of species under “optimal” growth conditions. As a minimum irradiance we used 5% (which is typical for a small gap) instead of 1%-2% of full irradiance that is typical for the forest understory, because many non-pioneer light demanding species were included in the study, and these would not have survived the understory light levels.

In another greenhouse (of about 15% irradiance) seeds were germinated in germination trays filled with sandy loamy soils. The soil was collected from a moist forest (Bobiri Forest Reserve). The soils were not acidic and had an average pH of 6.5 (± 0.28). The soils had moderate to high values of nitrogen, phosphorous, potassium and base saturation (see details of the chemical and physical properties of the soil in S1 Table). Germinated seeds were then grown in 9 cm wide by 20 cm long PVC (Polyvinyl chloride) tubes filled with sandy loamy soils (1130 cm^3^) collected from the moist forest. Sandy loam soil was preferred because it allows for good drainage, which is needed when plants are watered regularly. Seedlings were grown for three to four months before being transferred to the greenhouses. In the greenhouse, seedlings were allowed to acclimatise for four weeks before they were subjected to watering and no-watering treatments. We recorded daily temperature and relative humidity (S2 Table) over the period of the experiment using a Fisher memory hygrometer (Fisher Scientific, Pittsburgh, USA). The temperature and relative humidity values were used to estimate vapour pressure deficit in the greenhouses.

### Experimental design

The design in the greenhouse was a completely randomised factorial design with water, light and species as factors. We studied 96–112 individuals per species, which were randomly assigned to- and equally distributed amongst the four greenhouses; half of the individuals of each species in each greenhouse received water, and the other half were not watered for nine weeks. We simulated therefore not continuous low water conditions, but the effect of a prolonged dry season drought. Rainfall records taken from a rain gauge placed at 3 km from a dry semi-deciduous and a wet evergreen forest in the study region over a period of two years indicated a two-month period (December to January) in which there was no rain, especially in the dry forest. Therefore the nine weeks that water was withheld from seedlings in the greenhouse compares with the length of the dry season in the field. The experiment included in total 1,056 seedlings (6 species x 2 light treatments x 2 greenhouses x 2 drought treatments x 14 seedlings per treatment combination + (4 species x 2 light treatments x 2 greenhouses x 2 drought treatments x 12 seedlings per treatment combination). The positions of individual seedlings were rotated in the greenhouse every two weeks to ensure all species were exposed to the same environmental variation in the shade house. Because of limited seed availability and space constraints the experiment was conducted in two batches; the first was conducted from August-November 2010 and the second from February–May 2011. One additional pioneer species *(Ricinodendron heudelotii)* was included as a phytometer in both batches of the experiment to test for any systematic differences in the growing conditions in the greenhouses. A Mann Whitney U test of the differences in percent survival (in the dry treatment) between *Ricinodendron heudelotii* seedlings in the first and second batches of the experiment for low and high light treatments at the end of nine weeks did not show significant difference (Low light, Mann Whitney U = 39.50, Z = -0.11, P = 0.92, n = 19; high light, U = 40.0, Z = -0.05, P = 1.00, n = 19).

### Seedling performance and traits measurements

At the beginning and end of the experiment, randomly, eight individuals were selected for each species and their heights, diameters, leaf areas, stem and root lengths were measured and leaves counted. Leaves were digitised with a desk-top scanner (Canon Lide 100) and leaf area was determined with pixel counting software Image J [59]. Total root length was measured using the line intersect method [60]. Fresh weights of leaves, stems and roots were determined and the samples dried in an oven at 65°C for 48 hours. Relative growth rate was calculated for each species using initial and final harvest at the end of nine weeks using Equation 1 [61].

$$\text{RGR plant dry mass }=\;(\bar{\text{lnM}2}-\bar{\text{lnM}1)}/\left(\text{t}2-\text{t}1\right)$$ Equation 1

where $\bar{\text{ lnM}1}\;$ and $\bar{\text{lnM}2}$ are the means of natural logarithm transformed plant dry mass at time t_1_ and t_2._

The basic measurements were used to calculate nine seedling traits: leaf mass fraction (LMF; total leaf mass divided by plant mass, g g^-1^), stem mass fraction (SMF, stem mass divided by plant mass, g g^-1^), root mass fraction (RMF, root mass divided by plant mass, g g^-1^), specific leaf area (SLA, leaf area per leaf mass, cm^2^ g^-1^), leaf area ratio (LAR, total leaf area divided by plant mass, cm^2^ g^-1^), specific stem length (SSL, stem length divided by stem mass, cm g^-1^), specific root length (SRL, total root length divided by root mass, cm g^-1^), stem length per unit plant mass (SLPM, stem length divided by plant mass, cm g^-1^) and root length per unit plant mass (RLPM, total root length divided by plant mass, cm g^-1^). Leaf traits (LMF, SLA, and LAR), were calculated because of their importance for light capture, and stem traits (SSL and SLPM) because of their importance for vertical height expansion and hence, light capture [62], and for water transport and stability. The root traits (RMF, SRL and RLPM) were chosen because they are important for water capture [32], and for water storage (RMF).

### Assessment of survival, wilting and physiological measurements

Seedling survival was assessed every week. Seedling wilting stage was monitored following [63]. Mid-day leaf water potential (ψ_mid_) was measured for six slightly wilted individuals of each species in each greenhouse using the pressure bomb technique [64]. Stomatal conductance was measured on the same selected individuals using a leaf porometer (Model SC-1, Decagon Devices, USA). Similar measurements of stomatal conductance and leaf water potential were performed for the watered plants. Leaf water potential was measured because it gives an indication of the level of water stress in a plant and stomatal conductivity was chosen because it gives an indication of how plants control water loss through stomatal closure [19]. Drought survival in shade and in high light was quantified as the percentage of individuals alive in the dry low light- and dry high light treatments at the end of the experiment. The soil matric potential at which seedlings died in the dry treatment was estimated using the filter paper technique [65].

### Data analysis

A Mann-Whitney (exact test) was used to test the differences between drought survival after nine weeks under the dry treatment in the shade (5% irradiance) and in high light (20% irradiance). A three-way ANOVA was performed to evaluate the effects of species, light and water on relative growth rate and leaf physiology. Species, light and water were used as factors and RGR, leaf water potential and stomatal conductance were used as dependent variables. Greenhouses were not included as a factor in the final three-way ANOVA because the difference in the light treatment was large (5%-20%) and species values differed little between the two greenhouses belonging to the same light treatment. Data from the replicates of each light level was pooled together. A three-way ANCOVA was performed to evaluate the effects of species, light and water on morphological traits. Plant dry mass was included as a covariate to correct for ontogenetic effects due to variation in seedling size (cf. [62]). Allocation variables (LMF, SMF, and RMF) are proportional (between 0 and 1) and they were therefore arcsine transformed to enhance normality and to stabilise the variance. The other traits (SLA, SSL, SRL, LAR, SLPM, RLPM, stomatal conductance and leaf water potential) were log_10_ transformed to stabilise the variance. To analyse which individual species showed a significant response to light and water the AN(C)OVA’s were repeated for each individual species. Plasticity was calculated by first taking the average trait value of each of the four treatment combinations (dry and low light, dry and high light, wet and low light, wet and high light). Plasticity was then calculated for each trait as the difference between maximum mean value and minimum mean value of the treatment combinations, divided by the maximum mean value [44]. The relationship between overall plasticity index (mean of trait plasticity of the 9 individual traits) and growth under optimum conditions (high light, 20% irradiance and daily watering) was tested using Spearman correlation. Additionally, the relationship between overall plasticity index and survival under stressful conditions (high light, 20% irradiance and drought for nine weeks) was tested using a Spearman’s correlation. Plasticity of each of the 9 individual traits was also correlated with survival under stressful conditions and growth under optimal conditions, using the ten species as data points.

## Results

### Seedling survival and relative growth rate in response to drought and shade

**Fig 1:**
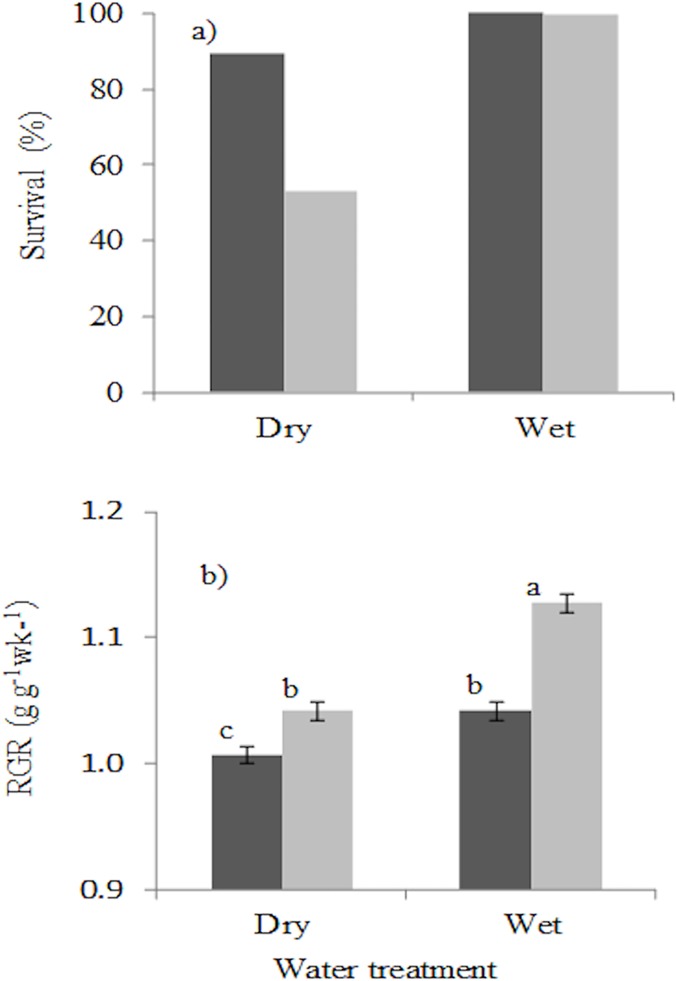
Performance of Ghanaian tree seedlings in terms of (a) survival and (b) relative biomass growth rate (RGR) in response to drought and shade. Shade treatments consisted of low light (5% of full sunlight, black bars) and high light (20% of full sunlight, grey bars). The water treatment consisted of a wet treatment (plants continuously watered for nine weeks) and dry treatment (water was withheld from seedlings for nine weeks). Means and standard error of the means are shown. Bars accompanied by a different letter are significantly different at P < 0.05 (ANOVA, Post hoc LSD test). n = 10 species.

Drought led to a decrease in survival compared to the continuously watered plant in both shade (89% drought survival versus 100% for continuously watered plants) and light (53% drought survival versus 99.6% for continuously watered plants). No statistical test was carried out to test the difference in survival in the watered plants because survival was 100% in the 5% irradiance greenhouse and 99.6% in the 20% irradiance greenhouse. There was a significant difference between drought survival in the shade (5% irradiance) and drought survival in the high light (20% irradiance) in the dry treatment (Mann-Whitney U = 10.5, *Z* = -2.99, P < 0.01). Drought survival in high light was 1.7 fold lower than in shade, indicating that plants are hit harder by drought in exposed environments that are typical of gaps (Fig 1A). Both drought and shade led to a decrease in relative biomass growth rate (Three-way ANOVA, drought, *P* ≤ 0.001; shade *P* ≤ 0.001; Fig 1B). There was a significant interaction effect (*P* ≤ 0.001) of light and water on relative growth rate, indicating that the effect of drought depended on the light level under which plants were growing. Drought reduced relative growth rate more strongly under high light than in low light.

### Drought and shade effects on allocation and morphology

Seedling responses were analysed with a three-way ANCOVA, with species, light, and water as factors, and plant dry mass at harvest as a covariate (Table 2). Overall the models explained a substantial part of the variation (average *R* ^*2*^: 0.78, *R* ^*2*^ range: 0.61–0.93). Biomass at final harvest did not have an effect on biomass allocation, but it had a strong effect on tissue morphology and whole-plant morphology (Table 2). Overall, species had the strongest effect on seedlings, affecting all traits, with high F values. Of the environmental factors, light had the strongest effect on seedlings, affecting 92% (11/12) of the traits evaluated. Water had a significant effect on 70% (7/10) of the traits evaluated, whereas there were considerably less significant interactions between light and water (40% of the cases, 4/10), and for those the *F* values were also much lower.

**Table 2:** A Three- way ANCOVA of species (S, df = 9), water (W, df = 1) and light (L, df = 1) as main factors and relative growth rate, biomass allocation, morphological and leaf physiological traits as dependent variables.

Traits	beta	Plant dry mass	Species	Light	Water	SX L	SXW	LXW	SX L XW	R^2^
Relative growth rate		ND	20.6***	81.9***	85.6***	0.9	6.3***	13.4***	0.45	0.92
Leaf mass fraction	0.02	0.8	105.7***	53.2***	2.5	7.1***	11.6	4.2*	4.0***	0.69
Specific leaf area	-0.15	33.1***	58.5	14.6***	ND	3.9	ND	ND	ND	0.66
Leaf area ratio	-0.14	17.5***	46.8***	34.5***	ND	3.9***	ND	ND	ND	0.69
Stem mass fraction	-0.02	1.5	190.5***	5.0*	3.4	5.6***	7.6***	3.9*	2.7***	0.77
Specific stem length	-0.62	1079.5***	67.9	50.9***	35.6	5.9	3.8	0.6	1.6	0.91
Stem length per unit plant mass	-0.64	1741. ***	275.5***	38.9***	10.7***	3.8***	5.0***	18.3	1.4	0.93
Root mass fraction	-0.01	0.2	52.2***	44.6***	5.7*	5.1***	5.1***	1.4	1.6	0.61
Specific root length	-0.33	79.2***	86.4***	16.6***	0.6	1.3	6.4***	3.9*	2.4*	0.69
Root length per unit plant mass	-.33	87.8***	113.5***	0.1	8.1*	1.3	10.2***	3.1	2.4*	0.77
Stomatal conductance	ND	ND	26.3***	72.9***	873.1***	6.4***	12.9***	1.9	3.4*	0.91
Leaf water potential	ND	ND	33.3***	10.4***	182.7***	4.8***	5.4***	0.0	3.1*	0.79

Plant dry mass was included in the ANOVA as a covariate for all variables but RGR, and beta is the regression coefficient of the slope. F-value, significance levels and R^2^ of the model are shown.

*:p ≤ 0.05

**: p ≤ 0.01

***: p ≤ 0.001

ND = not determined. Leaf mass fraction, stem mass fraction and root mass fraction were arcsine transformed and all other traits were log_10_ transformed prior to analysis.

### Drought effects on allocation and morphology

**Fig 2:**
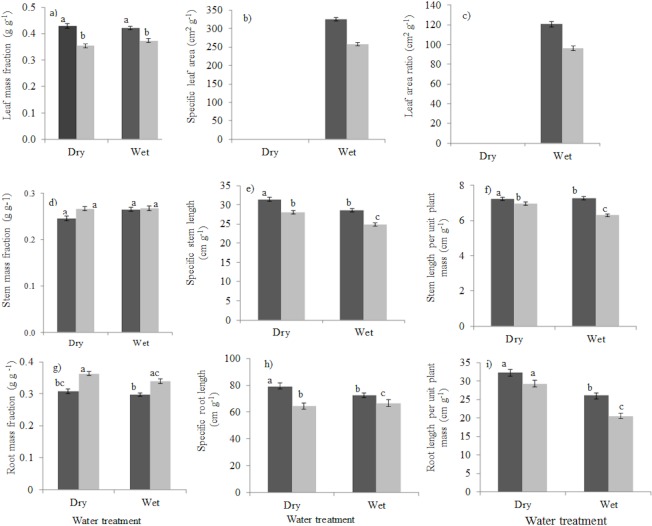
Seedling responses to drought and shade in terms of allocation (left panels), tissue morphology (middle panels) and whole-plant efficiency (right panels) for leaf- (top panels), stem- (middle panels) and root (lower panels) tissues. Shade consisted of low light (5% of full sunlight, black bars) and high light (20% of full sunlight, grey bars). The drought treatment consisted of a wet treatment (plants continuously watered) and dry treatment (water was withheld from plants for nine weeks). (a) leaf mass fraction (LMF), (b) specific leaf area (SLA), (c) leaf area ratio (LAR), (d) stem mass fraction (SMF), (e) specific stem length (SSL), (f) stem length per unit plant mass (SLPM), (g) root mass fraction (RMF), (h) specific root length (SRL), and (i) root length per unit plant mass (RLPM). Means and standard errors are shown. Bars accompanied by a different letter are significantly different (ANCOVA, Turkey’s test P<0.05). n = 10 species. For droughted plants no bars are shown for SLA and LAR, because the leaves were desiccated, and their leaf area could not be measured precisely.

Drought did not alter leaf mass fraction (*P* = 0.115; Fig 2A) which indicates that the transpiring leaf mass is not reduced under drought. The effects of drought on specific leaf area, and leaf area ratio could not be determined because leaves of seedlings were wilted at final harvest. There was no significant effect of drought on stem mass fraction (*P* = 0.066; Fig 2D) but drought led to an increase in specific stem length (*P* ≤ 0.001; Fig 2E) and stem length per unit plant mass (*P* ≤ 0.001; Fig 2F). Drought led to high allocation to roots (Root mass fraction, *P* = 0.018; Fig 2G), no significant change in specific root length (*P* = 0.423; Fig 2H) and a significantly higher root length per unit plant mass (*P* = 0.005; Fig 2I) which facilitates water uptake.

### Shade effects on allocation and morphology

Shade led to an increase in allocation to leaves (high leaf mass fraction, *P* ≤ 0.00; Fig 2A), increased production of thin and/or soft leaves (high specific leaf area, *P* ≤ 0.001; Fig 2B) and increased the leafiness of the seedlings (high leaf area ratio, *P* ≤ 0.001; Fig 2C) thus enhancing light capture. Shade led to a reduction in allocation to stem (low stem mass fraction, *P* = 0.025; Fig 2D), an increase in specific stem length (*P* ≤ 0.001; Fig 2E) and stem length per unit plant mass (*P* ≤ 0.001; Fig 2F). Shade decreased allocation to roots (low root mass fraction, *P* ≤ 0.001; Fig 2G) and increased investment in fine roots (high specific root length, *P* ≤ 0.001; Fig 2H). Hence, the lower investment in root biomass was compensated for by investment in a higher specific root length which resulted in a similar root length per plant mass for shaded plants compared to light plants (*P* = 0.817; Fig 2I).

### Combined impact of shade and drought on allocation and morphology

Forty percent (40%) of the variables showed an interaction of light and water, as demonstrated by ANCOVA analysis (Table 2). Relative growth rate (*P* ≤ 0.001), leaf mass fraction (*P* ≤. 05), stem mass fraction (*P* = 0.05) and specific root length (*P* = 0.048) were traits that responded to the combined effects of light and water. This indicates that relatively few variables responded to the combined effects of shade and drought. Drought reduced relative growth rate more strongly in high light than in low light and for stem mass fraction drought resulted in a stronger reduction in shade than in high light. For specific root length drought led to an increase in SRL in low light but a slight decrease in high light. Under drought leaf mass fraction was strongly reduced in shade compared to the wet treatment.

### The combined effects of shade and drought on individual species

To analyse how individual species responded to the combined effects of shade and drought a two-way AN(C)OVA was done per trait for each individual species. For most traits (9 out of 10 traits evaluated) there were significant interactions between shade and drought, but these were found for very few species (1 to 3 species per trait) (Table 3). This suggests that the interaction between species x shade and light in the three-way AN(C)OVAs (Table 2) was being accounted for by few species. It also indicates that the shade x drought interaction is species specific. Species that responded to the interactive effects of shade and drought were mostly non-pioneer light demanding species (*Albizia zygia*, *Pericopsis elata*, *Pouteria aningeri*, *Sterculia rhinopetala*, *Piptadeniastrum africanum*).

**Table 3:** A Two- way ANCOVA of individual species with water (W, df = 1) and light (L, df = 1) as main factors and relative growth rate (RGR), biomass allocation, morphological and leaf physiological traits as dependent variables.

Species	Factor	RGR	LMF	SMF	SSL	SLPM	RMF	SRL	RLPM	g	LWP
*Entandrophragma angolense*	PDM	ND	1.09	5.31*	181.90***	344.22***	0.99*	0.16	4.68*	ND	ND
	Light	21.90***	8.45**	0.56	0.20	2.09	10.88**	2.16	0.15		
	Water	5.59*	4.99*	0.74	0.52	0.33	1.56	0.242	0.01		
	L x W	5.17*	0.33	3.27	1.34	3.28	0.58	1.02	2.99		
*Turraeanthus africanus*	PDM	ND	11.29***	4.68*	29.22***	86.97***	2.52	2.25	5.60*	ND	ND
	Light	2.84	53.35***	8.46**	29.19***	0.99	18.01***	9.10**	0.87	9.73**	5.80*
	Water	0.01	9.9**	18.01***	3.30	19.15**	0.08	7.76**	7.99**	18.01***	18.01***
	L x W	0.98	0.12	0.08	3.944*	0.07	2.73	0.80	0.00	0.08	0.08
*Piptadeniastrum africanum*	PDM	ND	1.03	0.04	169.03***	211.75***	1.44	36.27***	38.79***	ND	ND
	Light	17.99***	2.83	2.70	11.46**	2.71	0.68	2.814	1.60	7.42*	0.13
	Water	20.95***	8.72**	0.90	15.30***	9.81**	14.24***	1.11	5.25*	95.24***	49.00***
	L x W	0.21	0.01	1.01	0.03	3.39	0.54	0.33	1.52	1.26	1.53
*Ceiba pentandra*	PDM	ND	0.05	1.04	126.92***	131.80***	2.5	10.55**	12.36**	ND	ND
	Light	13.71***	19.6***	2.69	50.43***	33.89***	23.3***	4.10*	5.52*		
	Water	141.52***	6.91*	7.43**	2.73	3.94*	0.17	0.08	0.37		
	L x W	1.39	0.08	1.47	5.80*	0.21	0.96	0.70	1.71		
*Albizia zygia*	PDM	ND	0.01	8.30	135.16***	171***	2.17	62.76***	41.15***	ND	ND
	Light	13.84***	0.77	0.01	8.08**	5.72*	1.15	0.03	0.01	11.79**	0.57
	Water	0.57	0.21	3.10	18.75***	3.15	1.56	37.26***	44.54***	160.85***	9.38**
	L x W	6.42	5.67*	0.36	0.76	1.92	7.40**	1.01	0.41	5.91*	4.24*
*Pericopsis elata*	PDM	ND	3.42	2.46	116.70***	451***	0.13	0.18	0.07	ND	ND
	Light	8.99**	0.76	0.99	5.55*	7.69**	0.29	2.84	3.85*	5.44*	4.46*
	Water	19.47***	0.71	1.79	8.92**	7.14*	8.63**	1.34	6.34*	279.28***	695.69***
	L x W	8.55**	**7.30** **	4.50*	1.20	3.64	0.23	1.30	0.74	7.26*	20.98***
*Sterculia rhinopetala*	PDM	ND	**2.00**	0.55	101.76***	145.01***	6.26*	25.39**	11.07**	ND	ND
	Light	6.92*	2.71	1.29	8.29**	1.60	1.29	3.40	1.36	1.31	3.95
	Water	7.07*	1.24	4.41*	15.80**	1.47	13.12**	0.92	5.19*	239.20***	5.41**
	L x W	0.79	0.02	0.25	2.08	9.71**	1.49	1.32	5.32*	0.13	0.36
(*Pouteria aningeri*)	PDM	ND	2.17	0.41	88.93***	99.77***	2.01	1.91	0.36	ND	ND
	Light	9.23**	0.70	0.52	0.73	0.00	0.19	1.24	0.88	89.14***	62.89***
	Water	13.76***	0.32	1.73	1.28	0.15	0.34	0.05	0.05	73.44	26.28***
	L x W	0.21	6.09*	2.80	1.17	2.47	3.41	0.33	0.26	5.14*	1.89
*Antiaris toxicaria*	PDM	ND	1.55	0.89	165.47***	204.09***	0.19	10.79**	15.47***	ND	ND
	Light	27.51***	0.97	11.65***	0.70	13.10***	6.94*	2.29	0.08		
	Water	8.71**	1.82	6.35*	0.47	6.45*	6.29*	1.38	0.03		
	L x W	0.21	0.10	0.19	0.13	0.28	0.03	1.35	2.32		
*Strombosia pustulata*	PDM	ND	2.72	0.95	47***	88.52***	6.99*	2.16	9.12**	ND	ND
	Light	27.49***	7.52**	0.35	1.54	1.22	7.34**	0.07	1.67		
	Water	23.05***	12.29**	3.96*	4.98*	0.34	6.31*	2.21	8.84**		
	L x W	6.21*	0.63	0.133	0.20	0.04	0.27	0.47	0.18		
Total number of light x water interactions		3	3	1	2	1	1	0	1	3	2

Plant dry mass (PDM) was included in the ANCOVA as a covariate for all variables but RGR. F-values and significance levels are shown.

*:p ≤ 0.05

**: p ≤ 0.01

***: p ≤ 0.001

ND = not determined. Leaf mass fraction (LMF), stem mass fraction (SMF) and root mass fraction (RMF) were arcsine transformed and all other traits were log_10_ transformed prior to analysis. SSL = specific stem length, SLPM = stem length per unit plant mass, SRL = specific root length, RLPM = root length per unit plant mass, g = stomatal conductance, LWP = Leaf water potential.

### Leaf physiology

**Fig 3:**
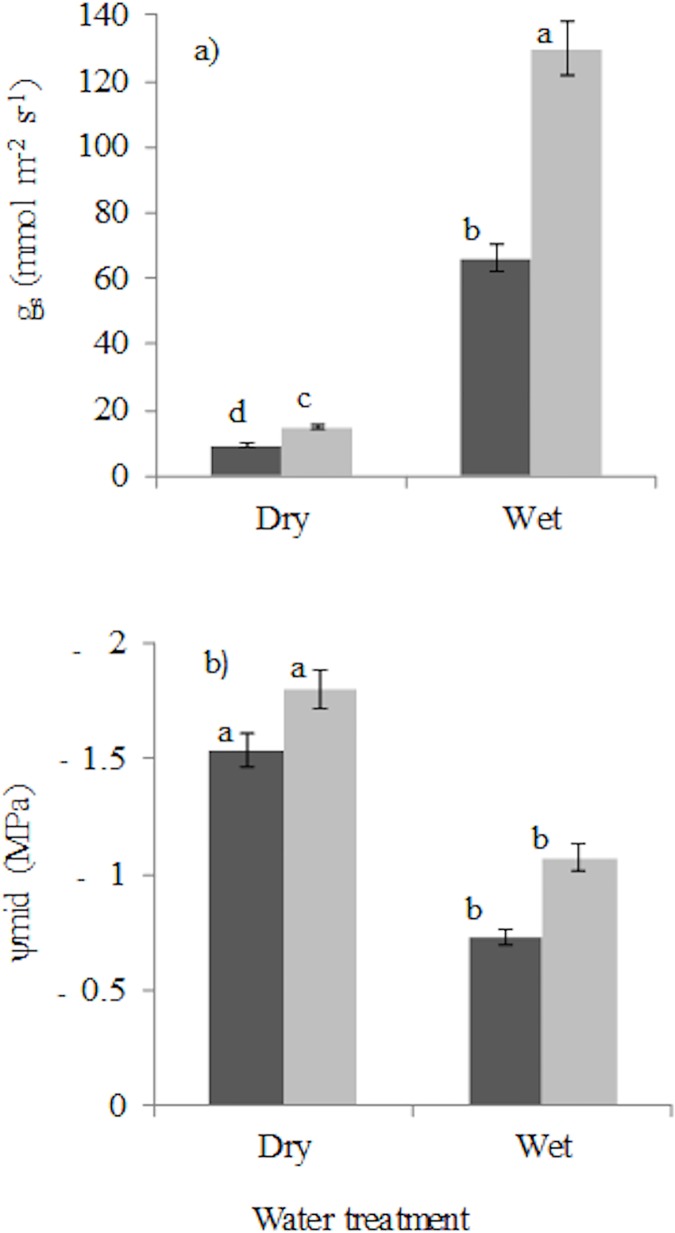
Seedling leaf physiological traits: (a) stomatal conductance (g_s_), (b) midday leaf water potential (ψ_mid_) of plants from which water was withheld and plants that received daily watering for 9 weeks in 5% of full sunlight (black bars) and 20% of full sunlight (grey bars). Bars are means and error bars are standard errors of the means.

Stomatal conductance and mid-day leaf water potential of slightly wilted seedlings showed significant response to drought and shade (Table 2). Drought led to a strong reduction (*P* ≤ 0.001) in stomatal conductance of the leaves that were slightly wilted (Fig 3A). Shade led to a reduction (*P* ≤ 0.001) in stomatal conductance (Fig 3A). Leaf water potential decreased in response to drought (*P* ≤ 0.001) but did not change with shade (*P* = 0.090) and there was no significant light x water interaction (*P* = 0.938; Fig 3B).

### The relationship between trait plasticity and species performance

**Fig 4:**
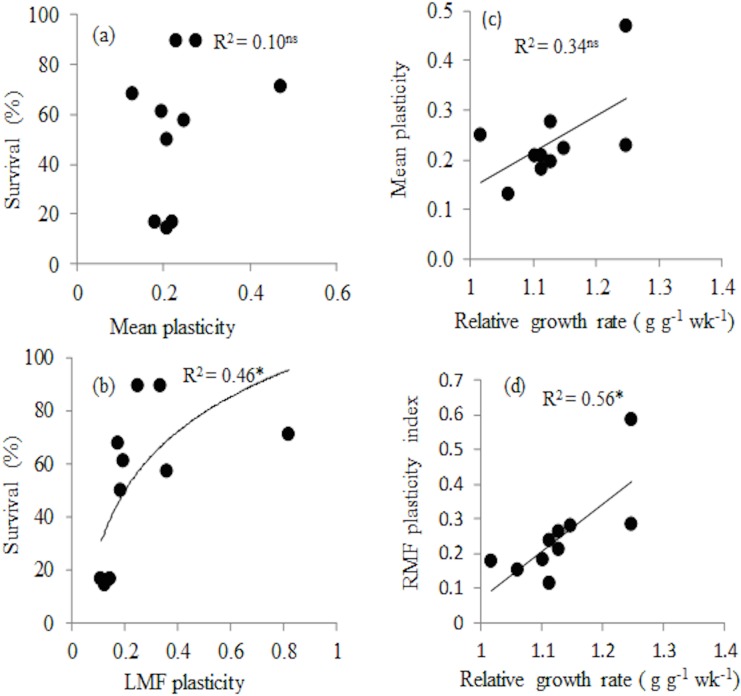
Relationship between survival under stressful conditions (drought and high light) and (a) mean plasticity of nine traits, (b) Leaf mass fraction (LMF) plasticity. Relationship between relative growth rate under optimal conditions (continuous watering and 20% of full sunlight) and (c) mean plasticity and (d) root mass fraction plasticity. Plasticity for each trait was calculated as maximum minus minimum mean trait values divided by maximum mean trait values across four treatment combinations. For each species, mean plasticity was calculated as the average plasticity of 9 traits. Regression line and coefficient of determination are shown. Ns = not significant, * = p ≤ 0.05, ** = p ≤ 0.01, n = 10 species.

To evaluate whether trait plasticity was associated with seedling performance correlations were tested between mean plasticity (mean plasticity across nine traits) and RGR under optimal conditions (wet treatment in 20% light), between mean plasticity and survival under stressed condition (dry treatment in 20% light) and between plasticity in RGR and survival. RGR under optimal conditions was only significantly correlated with plasticity in RMF (*r* = 0.81, P ≤ 0.01; Table 4, Fig 4D). Survival under stressful conditions was only significantly and positively correlated with plasticity in leaf mass fraction (Fig 4B, *r* = 0.74, P = 0.014). Plasticity in survival was significantly negatively related to plasticity in leaf mass fraction (r = -0.71, P = 0.023). Plasticity in RGR was not significantly related to plasticity of any of the traits (Table 4).

**Table 4:** Spearman rank correlation between plasticity in functional traits and seedling performance of 10 Ghanaian tree species; their relative growth rate under optimal conditions (20% of full sunlight and nine weeks watering), plasticity in relative growth rate, survival under stress conditions (no watering for 9 weeks in 20% of sunlight), plasticity in stress survival, and mean plasticity across four treatment combinations (5% of sunlight and no watering, 20% of sunlight and no watering, 5% of sunlight and watered, and 20% of sunlight and watered).

Plasticity trait	RGR (optimum)	Plasticity RGR	Stress survival (%)	Plasticity survival
Leaf mass fraction	0.19	0.13	0.74*	-0.71*
Stem mass fraction	-0.08	0.08	-0.45	0.61
Root mass fraction	0.81**	0.54	0.32	-0.32
Specific leaf area	-0.07	-0.15	-0.52	0.46
Specific stem length	0.13	0.16	0.01	-0.20
Specific root length	0.59	-0.14	0.56	-0.43
Leaf area ratio	0.07	-0.16	0.02	-0.07
Stem length per unit plant mass	0.62	0.19	0.35	-0.37
Root length per unit plant mass	0.58	-0.02	0.53	-0.49
Overall mean plasticity	0.47	-0.03	0.43	-0.38

n = 10 species. Significance levels of the correlation are shown.

*: p ≤ 0.05

***: P ≤ 0.001

RGR = Relative Growth Rate.

## Discussion

### Seedling performance in response to drought and shade

We hypothesized that drought and shade would reduce seedling growth and survival because of a lower resource availability (the *resource limitation hypothesis*), and that drought survival would be higher under shade than in high light because of improved microclimatic conditions (the *facilitation hypothesis*). Our results are in line with both hypotheses (Fig 1). The weaker impact of drought in shade is consistent with the facilitation hypothesis [24], and other studies in which drought sensitive seedlings growing under herbaceous layer in Californian Chaparral had high survival [26,66]. Under shaded conditions in the field, plants are exposed to low air temperatures and low vapour pressure deficits resulting in less drought stress and enhanced drought survival [cf. 24, 26, 67]. Yet, in our study the temperature, relative humidity and vapour pressure deficit did not differ significantly between the greenhouses with 5 and 20% irradiance (S2 Table). The higher drought survival in the low light greenhouse can therefore be attributed to the soil water reserve in the pot being depleted more slowly because of a combination of 1) smaller seedlings (Fig 1B) with smaller transpiring leaf area, 2) lower transpiration rates per unit leaf area (Fig 3A) and 3) less water evaporation from the soil. The soil matric potential at which individual species died in the two treatments did not differ significantly for more than half (5 out of 8) of our study species (S3 Table). Therefore though there was some difference in soil water availability in the pots for some species in the two treatments, it had a less effect on the overall effectiveness of the drought treatment.

Our results contrast with the *trade-off hypothesis* [22], which states that drought should have *stronger* impact in shade than in high light, because shaded plants invest relatively less in roots, and have therefore less access to soil water. We indeed found that shaded plants had lower RMF, in line with the *trade-off hypothesis* [22], but they compensated for this with a higher specific root length, resulting in a similar root length per plant mass, and hence capacity for water uptake, as high-light plants (Fig 2H and 2I).

Under field conditions an enhanced survival below shaded tree crowns can also result from an increased nutrient input by tree litter [23]. In the understory of tropical forests, saplings growing under tree crowns invest in long-lived leaves which are characterized by high levels of defense against herbivores [68]. Other studies found contrasting results; drought survival in the understory was lower compared to plants growing in the centre of a forest gap [19] in West Africa in line with the trade-off hypothesis. Such findings could partly be attributed to the fact that plants in shade suffer from competition for water uptake by canopy trees [69]. Even under roots competition some shaded plants in the field have been found to survive drought [85]. Such species invest in relatively high allocation to roots in addition to investment in thick, though, long-lived leaves [85]. The enhanced drought survival in shade that we found might allow these species to persist in the forest understory when seasonal drought increases over time, as has been observed in the tropical forest zone of Ghana [86]. We should emphasize that this is a greenhouse study where plants were grown alone in a limited soil volume. Under field conditions shaded understory plants may suffer from strong competition for water by canopy trees. In the field, other drought-coping mechanisms might become important as well. For instance plants growing in high irradiance in the field will take advantage of their fast growth and large size at the end of the wet season. As a result, they can forage during the dry season a larger soil volume, or deeper soil layers for water, where it is more readily available [26, 70].

Both drought and shade reduced relative growth rate which is in agreement with our *resource limitation* hypothesis, and consistent with other studies of Mediterranean species growing in the field or under controlled conditions [25, 26, 46]. We found that shade reduced RGR more strongly in wet- compared to dry conditions, which contrast with the findings of other studies [25, 26] that reported proportional reduction of RGR in low light and high light (*i*.*e*., no interaction effect).

### Morphological and physiological response of seedlings to drought

We hypothesized that plants will invest in the organ that captures the most limiting resource (*Brouwer’s hypothesis*). Generally species growing in drier environment may improve water capture through investment of more biomass in roots and the production of thin roots with high specific root length (SRL) and an increase in root length per unit plant mass (RLPM) at the whole plant level [32]. We indeed found that under drought seedlings had higher RMF and RLPM, in line with *Brouwer’s hypothesis*, although they did not produce roots with high SRL (Table 2; Fig 2G and 2I). In a study of thirteen temperate species, reduction in watering led to an increase in RMF [26] but in another study of four temperate shade-tolerant species no significant difference in LMF, SMF and RMF were found across watering treatments [25]. Roots are, apart from water uptake, also important for water storage. For example, seedlings of Baobab provenances from drier areas had higher RMF [71], which allowed them to store more water, and realize higher conductance and photosynthetic rates during drought.

In our study, drought led to increased drought stress as indicated by more negative mid-day leaf water potentials, and lower stomatal conductance (Fig 3A). The lower stomatal conductance implies reduced assimilation rate, (cf.[72–74]) which also explains the reduced growth of droughted plants (Fig 1). There is a continuum of stomatal responses to drought which ranges from drought avoidance, in which the stomata close at a threshold water potential to reduce transpiration and cavitation, to drought tolerance in which stomatal control is less severe and leads to higher transpiration rate [75, 76].

Plant may also reduce water loss during drought by reducing biomass fraction in transpiring leaves. This strategy was not confirmed in our study, as there was no significant change in leaf mass fraction in response to drought. We could not evaluate the effects of drought on SLA and LAR (see methods) but other studies found that seedlings that grow under dry conditions have low SLA and LAR which help to reduce water loss through transpiration [32]. In another study, water stress did not have an effect on SLA [74].

### Morphological and physiological response of seedlings to shade

In low irradiance light is a limiting resource, and shaded seedlings had higher LMF, SLA and LAR and, hence, higher light capture, in line with *Brouwer’s hypothesis* (cf.[77–79]). Shaded plants invested less biomass in stems, which could curtail their ability to overtop neighbours and access light. Yet, they compensated for this by producing slender stems with high specific stem length, leading to a higher stem length per unit plant mass (Fig 2E and 2F). Such an etoliation response to shading has also been found for other tree seedlings [62].

The increased biomass allocation to leaves in the shade came not only at the expense of a reduced biomass allocation to stem, but also at the expense of reduced biomass allocation to roots (Fig 2G). This is seemingly in line with the *trade-off hypothesis*, which states that an increase in shade tolerance comes at the expense of an increase in drought tolerance [22]. Yet, shaded plants compensated for a reduced RMF by producing fine roots, leading to similar root length per unit plant mass, and hence, to a similar ability to capture water. The higher LAR and SLA of shaded plants could potentially lead to a higher evaporative load, but this is thought to be compensated for by low air temperature and vapour pressure deficit in shade [25]. However in our study, air temperatures and vapour pressure deficit did not significantly differ between shade and high light treatments. Leaf water potential was more negative in high light compared to the shade (Fig 3B), indicating that high-light plants are more water-stressed probably because of high demand for water as a result of higher growth rate. At the same time the stomatal conductance was higher, indicating that stomata are also sensitive to the amount of light. The high stomatal conductance facilitates higher assimilation rates, and therefore faster growth rates in high-light compared to shaded plants (Fig 1). The results of the study is consistent with findings of a study conducted in South-western Spain in which both watered and droughted seedlings showed higher stomatal conductance under high light conditions [74].

### Combined effects of shade and drought on plant

Our study results showed within species performance trade-off between survival and growth as seedlings growing in the shade in drought had high survival but low relative growth rate compared to seedlings growing in high light, which is in agreement with the growth-survival trade-off found for seedlings and saplings across tropical tree species [69, 80]. A trade off in biomass allocation was found in our study as plants in shade allocated more biomass to leaves and plant in drought allocated more biomass to roots. Similar trade-offs have been found for other studies across 806 temperate tree species [81] and across tropical species of Bolivia [32] where seedlings of species from drier forest allocated more biomass to roots, whereas seedlings of species from moist forest allocated more biomass to leaves. Although our study focused on intraspecific responses compared to the other studies mentioned here, we make these comparisons because intraspecific acclimatisation found within species parallel adaptation responses found across species. For most of the traits we studied there was no significant interaction between shade and drought. When individual species responses were analysed, then for most of the traits 1–3 species showed significant interactive effects between shade and drought (Table 3). This indicates that drought and shade tolerance can vary independently for most of the species but not for others. Seedlings were able to tolerate combined shade and drought probably as a result of investment in organs that help to reduce demand for resources [30]. Our leaf water potential data suggest that shade alleviates drought impacts (although we did not find a significant shade x drought interaction) which contrasts sharply with the results of another study that reported a greater decline of leaf water potential with drought stress in the shade than in the sun [82]. Perhaps this is because the study was carried out in an unusually dry El Niño year in Central California.

### Relationship between plant trait plasticity and seedling performance

We hypothesized that higher plasticity in plant functional traits will lead to high survival under stressed conditions [45]. This hypothesis was rejected, as overall plasticity index across our treatment combination was not significantly related to survival under stressful conditions (Fig 4A). A study on seedlings of Mediterranean woody species neither found a relationship between overall phenotypic plasticity in response to water availability and enhanced performance under drought [41]. Plasticity in traits is not always an adaptive feature of plants, but in situation where it is considered adaptive feature it should lead to higher survival under stressful condition [45]. In our study, plasticity in leaf mass fraction was significantly related to survival under stressful conditions (Fig 4B). Perhaps the plasticity of individual key traits is more important in influencing species survival than a combination of traits. Adjusting the transpiring leaf mass may be an important mechanism to adjust to reduced water availability. Deciduousness, for example, has been found to be one of the determinants of drought survival [32]. A positive correlation between percent leaf loss and drought survival has also been found among individuals across three evergreen species in Australia [83].

We also predicted that faster growth under optimal condition would lead to higher plasticity [43, 44] because under high resource availability more carbon is produced and can be invested for traits adjustments in response to changing environment (cf.[43–44]). Yet, fast growth was not related with a higher overall plasticity. It was related with a high RMF. The ability of plants to show plastic responses to environment may in part define the ecological niche width of species [84] and species with higher plasticity in water balance related traits will more likely acclimatise to increased frequency of drought in many tropical forest areas.

## Conclusions

Shaded plants in the greenhouse survive drought better than plants in high light but they do so at the expense of their relative growth rates. Drought and shade had no interactive effects on majority of our study species. Our results suggest that shaded tropical forest species are potentially able to survive under prolonged droughts, but it should be checked whether these results also hold in the field where shaded understorey plants can grow in an unlimited soil volume and face competition for water by large canopy trees. A greater percentage (60%) of traits studied responded independently to drought and shade which allows for niche differentiation under any combination of water availability and shade. Overall traits plasticity was not related to survival under stressed condition though plasticity in leaf mass fraction was significantly related to survival under stressful conditions. Plasticity of individual traits that are associated with water balance may play a role in how seedlings survive under drought and shade.

## Supporting Information

S1 Dataset S1 Dataset contains data on leaf, stem and root traits, leaf water potential, stomatal conductance, plasticity index, relative growth rate, environmental variables and soil matric potential. (XLSX)

S1 Table S1 Table contains the chemical and physical properties of the soil used for the experiment. (DOCX)

S2 Table S2 Table contains the mean temperature, relative humidity and vapour pressure values in the greenhouses over the period of the experiment. (DOCX)

S3 Table S3 Table contains the mean soil matric potential values at which mortality of individual species occurred. (DOCX)
