# Supplementary material for: The Effects of Drought and Shade on the Performance, Morphology and Physiology of Ghanaian Tree Species
Source: PLoS One. 2015 Apr 2;10(4):e0121004. doi: 10.1371/journal.pone.0121004 (PMC4383566; doi:10.1371/journal.pone.0121004)
Supplement: S1 Table — (DOCX) [file pone.0121004.s002.docx]

S1Table. Acidity and nutrient concentrations of moist forest soil used in the drought and shade experiment. Means and standard deviation are shown.

| Acidity/Nutrients | Mean | Standard Dev. | indication |
| --- | --- | --- | --- |
| pH | 6.5 | 0.28 | neutral |
| Organic Carbon (%) | 2.54 | 0.41 |  |
| Organic Matter (%) | 4.38 | 0.03 | moderate |
| Exchangeable Ca (meq/100g) | 11.24 | 2.21 | high |
| Exchangeable Mg (meq/100g) | 3.26 | 1.057 | low |
| Exchangeable K (meq/100g) | 0.99 | 0.23 | high |
| Cation Exchange Capacity (meq /100g) | 15.67 | 3.56 | moderate |
| Base Saturation (%) | 99.33 | 0.17 | high |
| Phosphorus (ppm) | 7.44 | 1.18 | low |
| Potassium (ppm) | 128.61 | 23.97 | high |
