# Supplementary material for: The Effects of Drought and Shade on the Performance, Morphology and Physiology of Ghanaian Tree Species
Source: PLoS One. 2015 Apr 2;10(4):e0121004. doi: 10.1371/journal.pone.0121004 (PMC4383566; doi:10.1371/journal.pone.0121004)
Supplement: S2 Table — (DOCX) [file pone.0121004.s003.docx]

| Environmental variable | Experiment batch | Mean (%) (±SE) | F | P | df |
| --- | --- | --- | --- | --- | --- |
| Temperature | Batch 1 | H = 27.95(0.12) | 0.14 | 0.710 | 1 |
|  |  | L = 28.0(0.12) |  |  |  |
| Relative humidity | Batch 1 | H = 76.1(0.53) | 1.69 | 0.191 | 1 |
|  |  | L = 77.02 (0.53) |  |  |  |
| Vapour pressure deficit | Batch 1 | H = 0.90 (0.001) | 0.80 | 0.372 | 1 |
|  |  | L = 0.86 (0.001) |  |  |  |
| Temperature | Batch 2 | H = 30.33(0.11) | 1.83 | 0.177 | 1 |
|  |  | L = 30.53(0.11) |  |  |  |
| Relative humidity | Batch 2 | H = 65.6(0.65) | 0.23 | 0.629 | 1 |
|  |  | L = 65.19(0.65) |  |  |  |
| Vapour pressure deficit | Batch 2 | H = 1.51 (0.0001) | 0.10 | 0.747 | 1 |
|  |  | L = 1.50 (0.0001) |  |  |  |
| Temperature | Overall | H = 29.0(0.11) | 0.71 | 0.399 | 1 |
|  |  | L = 29.17(0.11) |  |  |  |
| Relative humidity | Overall | H = 71.26 (0.52) | 0.19 | 0.665 | 1 |
|  |  | L = 71.57 (0.52) |  |  |  |
| Vapour pressure deficit | Overall | H = 1.15 (0.00) | 0.10 | 0.750 | 1 |
|  |  | L = 1.14 (0.00) |  |  |  |

S2 Table. Temperature (^o^C.), relative humidity (%) and vapour pressure deficit in the four greenhouses (High light 1 and 2 (H) = 20% sunlight; Low light 1 and 2 (L) = 5% sunlight in the two experimental batches (1 & 2) over the experimental period of three months, which includes the four weeks that seedlings were conditioned in the greenhouses. Means and standard errors are shown. Data for the two green houses in each light treatment were pooled together for the analysis as there were not significant differences in the data of the two greenhouses under each light level. Temperature batch 1 df (error) = 338, batch 2, df (error) = 288; Relative humidity batch 1 df (error) = 338, Batch 2 df (error) = 288; Vapour pressure deficit batch 1 df (error) = 338, Batch 2 df (error) = 286; overall temperature df (error) = 628; Overall relative humidity, df (error) = 628; overall vapour pressure deficit, df (error) = 626.
