# Supplementary material for: The Effects of Drought and Shade on the Performance, Morphology and Physiology of Ghanaian Tree Species
Source: PLoS One. 2015 Apr 2;10(4):e0121004. doi: 10.1371/journal.pone.0121004 (PMC4383566; doi:10.1371/journal.pone.0121004)
Supplement: S3 Table — (DOCX) [file pone.0121004.s004.docx]

| Species | Mean (%) | SE (±) | F | P | df error |
| --- | --- | --- | --- | --- | --- |
| *Entandrophragma angolense* | H = -84.70 | 27.2, -39.9 | 5.80 | 0.027 | 18 |
|  | L = -22.28 | 7.40, -10.8 |  |  |  |
| *Turraeanthus africanus* | H = -91.68 | 7.2, -7.8 | 0.00 | 0.997 | 18 |
|  | L = -91.68 | 7.2, -7.8 |  |  |  |
| *Piptadeniastrum africanum* | H = -119.50 | 2.7, -2.8 | 0.24 | 0.632 | 18 |
|  | L = -117.58 | 2.7, -2.8 |  |  |  |
| *Ceiba pentandra* | H = -87.51 | 31.9, -49.9 | 6.97 | 0.018 | 16 |
|  | L = -14.14 | 6.0, -9.8 |  |  |  |
| *Sterculia rhinopetala* | H = -46.42 | 4.1,- 4.5 | 25.95 | 0.00 | 18 |
|  | L = -24.00 | 2.2, -2.4 |  |  |  |
| *Aningeria robusta* (*Pouteria aningeri*) | H = -43.67 | 3.3, - 3.5 | 0.03 | 0.869 | 18 |
|  | L = -42.85 | 3.2, -3.5 |  |  |  |
| *Antiaris toxicaria* | H = -85.30 | 8.9, -9.9 | 0.807 | 0.381 | 17 |
|  | L = -93.43 | 10.1, -11.3 |  |  |  |
| *Strombosia pustulata* | H = -61.95 | 7.7, - 8.8 | 2.345 | 0.143 | 18 |
|  | L = -46.75 | 5.9, - 6.7 |  |  |  |
| **Albizia zygia* |  |  |  |  |  |
| **Pericopsis elata* |  |  |  |  |  |

S3 Table. Mean and ± SE of soil matric potential at which mortality occurred in individual species in the drought treatment under 20% (H) and 5 % (L) light, F values, P values and the degree of freedom (df) of the error term. Results of a one-way ANOVA of the effects of light on the soil water content at which mortality occurred in individual species. * These species did not have mortality in the 5% light treatment at the end of the experiment and were excluded from the analysis.
